# Supplementary material for: Temperature and species-dependent regulation of browning in retrobulbar fat
Source: Sci Rep. 2021 Feb 4;11:3094. doi: 10.1038/s41598-021-82672-9 (PMC7862600; doi:10.1038/s41598-021-82672-9)
Supplement: Supplementary file 1 — Supplementary Figure Legends. [file 41598_2021_82672_MOESM1_ESM.docx]

**Figure S1. Mouse orbital fat derives from Wnt1-positive precursors, but other murine fat depots do not. A-C.** Immunohistochemistry of paraffin section of orbital soft tissue from a *Wnt1:Cre; Ai9* mouse reveals the presence of tdTomato staining of orbital fat. Arrows indicate examples of cells expressing tdTomato. **D-F.** Immunohistochemistry of paraffin section of interscapular brown fat from a *Wnt1:Cre; Ai9* mouse shows no tdTomato staining. **G-I.** Immunohistochemistry of paraffin section of inguinal fat from a *Wnt1:Cre;Ai9* mouse reveals weak and diffuse tdTomato staining that is not localized to adipocytes. **J-L.** Immunohistochemistry of paraffin section of epididymal fat from a *Wnt1:Cre; Ai9* mouse reveals the absence of tdTomato staining. **M-O.** Immunohistochemistry of paraffin section of perirenal fat from a *Wnt1:Cre; Ai9* mouse shows no tdTomato staining. **P-R.** Immunohistochemistry of paraffin section of retroperitoneal fat from a *Wnt1:Cre; Ai9* mouse shows no tdTomato staining. Scale bars =50 μm.

**Figure S2. RNA-Seq analysis of extraocular muscles identifies contaminating transcripts in orbital fat samples. A.** Principal component analysis of transcriptome data from mouse brown fat (BF), epididymal fat (EF), inguinal fat (IF), orbital fat (OF) and extraocular muscle (EOM) demonstrates that BF and OF segregate more closely to each other than to EF and IF. EOM segregates separately from all fat depts. **B.** Sample-wise correlation of transcriptome data from BF, EF, IF and OF demonstrates that transcriptome of OF is more closely correlated with that of BF than with the transcriptomes of EF, IF, and EOM. **C.** Heat map of transcriptome data from mouse BF, EF, IF, OF, and EOM demonstrating that there is little overlap between EOM and OF. Heatmap was created using Matlab 2018.

**Figure S3. Mouse orbital fat co-expresses *Cxcl12* and brown fat markers.** **A.** smFISH demonstrates colocalization of expression of *Cxcl12* and *Prdm16*. **B-D.** Higher magnification of A. Arrows indicate examples of cells with coexpression of *Cxcl12* and *Prdm16*. **E.** smFISH demonstrates colocalization of expression of *Ppargc1a* and *Prdm16*. **F-H.** Higher magnification of E. Arrows indicate examples of cells showing coexpression of *Ppargc1a* and *Prdm16*. Scale bars A, B = 50 μm. Scale bars, C-H = 10 μm.
