# Supplementary material for: Temperature and species-dependent regulation of browning in retrobulbar fat
Source: Sci Rep. 2021 Feb 4;11:3094. doi: 10.1038/s41598-021-82672-9 (PMC7862600; doi:10.1038/s41598-021-82672-9)
Supplement: Supplementary file 2 — Supplementary Figures. [file 41598_2021_82672_MOESM2_ESM.pdf]

Supplemental Information for:

Temperature and species-dependent regulation of browning in retrobulbar fat.

Fatemeh Rajaii, Dong Won Kim, Jianbo Pan, Nicholas R. Mahoney, Charles G. Eberhart, Jiang Qian, and Seth Blackshaw

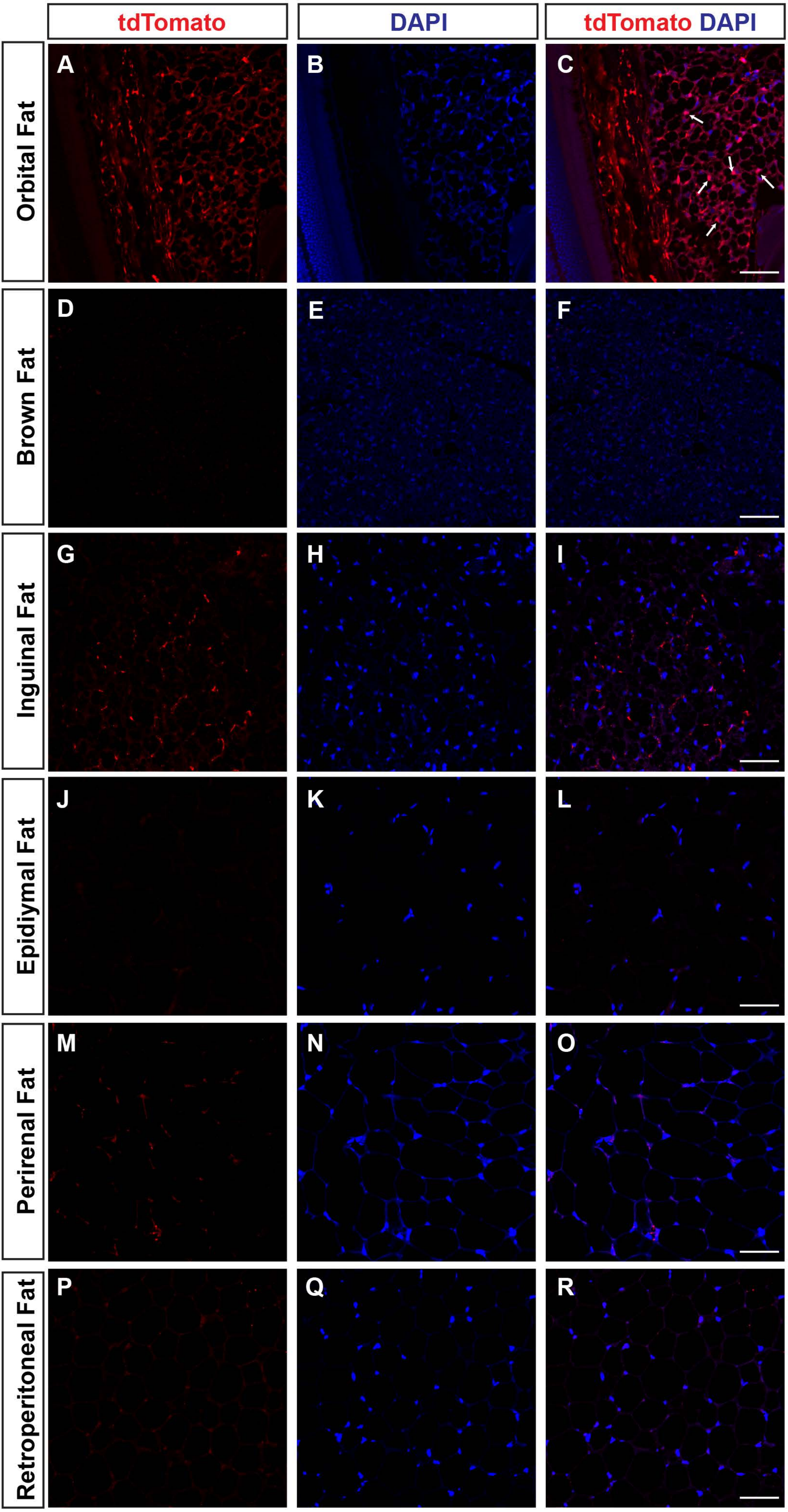

Rajaii, et al. Fig. S1

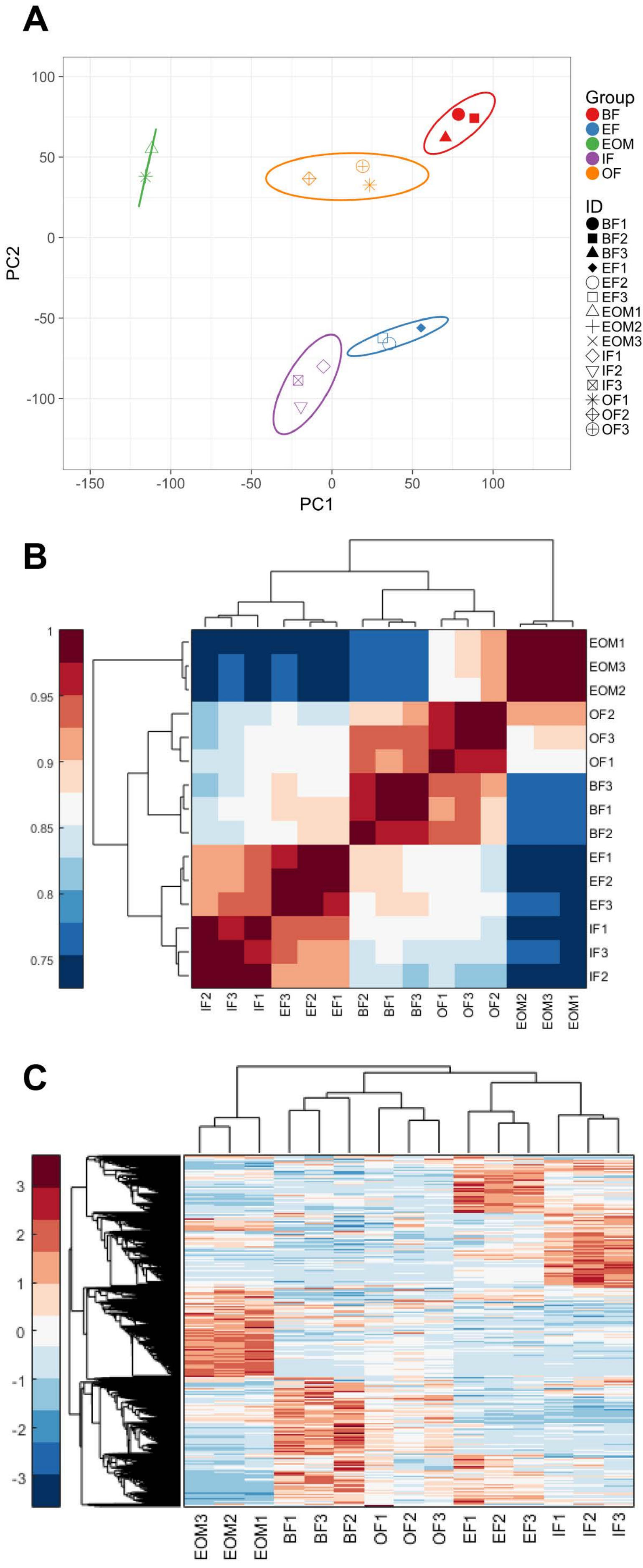

Rajaii, et al. Fig. S2

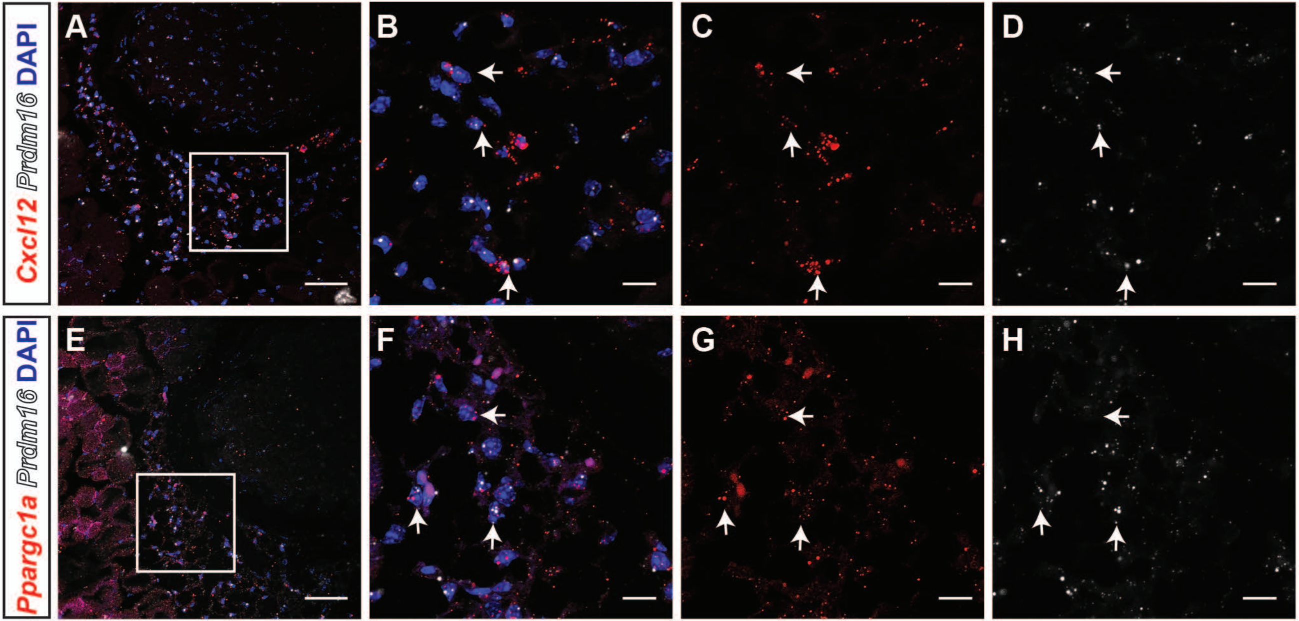

Rajaii, et al. Fig. S3
